# Patient Experience in Virtual Visits Hinges on Technology and the Patient-Clinician Relationship: A Large Survey Study With Open-ended Questions

**DOI:** 10.2196/18488

**Published:** 2021-06-21

**Authors:** Susannah Rose, Heather McKee Hurwitz, Mary Beth Mercer, Sabahat Hizlan, Kari Gali, Pei-Chun Yu, Caroline Franke, Kathryn Martinez, Matthew Stanton, Matthew Faiman, Peter Rasmussen, Adrienne Boissy

**Affiliations:** 1 Office of Patient Experience, Clinical Transformation Cleveland Clinic Cleveland, OH United States; 2 Taussig Cancer Center Cleveland Clinic Cleveland, OH United States; 3 Digital Health Cleveland Clinic Cleveland, OH United States; 4 Quantitative Health Sciences Cleveland Clinic Cleveland, OH United States; 5 Center for Value-Based Care Research Cleveland Clinic Cleveland, OH United States

**Keywords:** telehealth, virtual visit, patient experience, mobile phone

## Abstract

**Background:**

Patient satisfaction with in-person medical visits includes patient-clinician engagement. However, communication, empathy, and other relationship-centered care measures in virtual visits have not been adequately investigated.

**Objective:**

This study aims to comprehensively consider patient experience, including relationship-centered care measures, to assess patient satisfaction during virtual visits.

**Methods:**

We conducted a large survey study with open-ended questions to comprehensively assess patients’ experiences with virtual visits in a diverse patient population. Adults with a virtual visit between June 21, 2017, and July 12, 2017, were invited to complete a survey of 21 Likert-scale items and textboxes for comments following their visit. Factor analysis of the survey items revealed three factors: experience with technology, patient-clinician engagement, and overall satisfaction. Multivariable logistic regression was used to test the associations among the three factors and patient demographics, clinician type, and prior relationship with the clinician. Using qualitative framework analysis, we identified recurrent themes in survey comments, quantitatively coded comments, and computed descriptive statistics of the coded comments.

**Results:**

A total of 65.7% (426/648) of the patients completed the survey; 64.1% (273/426) of the respondents were women, and the average age was 46 (range 18-86) years. The sample was geographically diverse: 70.2% (299/426) from Ohio, 6.8% (29/426) from Florida, 4.2% (18/426) from Pennsylvania, and 18.7% (80/426) from other states. With regard to insurance coverage, 57.5% (245/426) were undetermined, 23.7% (101/426) had the hospital’s employee health insurance, and 18.7% (80/426) had other private insurance. Types of virtual visits and clinicians varied. Overall, 58.4% (249/426) of patients had an on-demand visit, whereas 41.5% (177/426) had a scheduled visit. A total of 41.8% (178/426) of patients had a virtual visit with a family physician, 20.9% (89/426) with an advanced practice provider, and the rest had a visit with a specialist. Most patients (393/423, 92.9%) agreed that their virtual visit clinician was interested in them as a person, and their virtual visit made it easy to get the care they needed (383/421, 90.9%). A total of 81.9% (344/420) of respondents agreed or strongly agreed that their virtual visit was *as good as* an in-person visit by a clinician. Having a prior relationship with their virtual visit clinician was associated with less comfort and ease with virtual technology among patients (odds ratio 0.58, 95% CI 0.35-0.98). In terms of technology, patients found the interface easy to use (392/423, 92.7%) and felt comfortable using it (401/423, 94.8%). Technical difficulties were associated with lower odds of overall satisfaction (odds ratio 0.46, 95% CI 0.28-0.76).

**Conclusions:**

Patient-clinician engagement in virtual visits was comparable with in-person visits. This study supports the value and acceptance of virtual visits. Evaluations of virtual visits should include assessments of technology and patient-clinician engagement, as both are likely to influence patient satisfaction.

## Introduction

Virtual visits have emerged at a time in health care when clinicians’ decisions have become only one part of patients’ experiences. Patients’ and their families’ needs, values, and inputs influence care decisions [1]. Patients are researching treatments on the internet and engaging as advocates and even as partners with physicians both on the internet and in person [1-5]. A crucial aspect of patient experience is the patient-clinician engagement or using a *relationship-centered care* approach. This approach prioritizes how the communication and emotions shared between patients and clinicians gradually affect patients’ experiences as a whole person [5,6]. Furthermore, whether physicians empathize with their patients strongly shapes the patient-physician relationship and patient satisfaction [7]. Virtual visits must continue the best aspects of in-person care to be an acceptable substitute for in-person care. However, no studies have evaluated whether virtual visits are addressing patients’ experiences comprehensively rather than simply whether or not they are satisfied.

Studies on virtual visits have examined patient satisfaction. For example, patients have reported positive experiences about communication, engagement, and building rapport with clinicians during their virtual visits [8-12]. Furthermore, patients have expressed that virtual visits are convenient in decreasing travel time, wait time, and stress and increasing comfort and convenience [13-15]. Virtual visits increased substantially during the initial phase of the COVID-19 pandemic, and physicians reported that patients respond positively to virtual visits [16], that telemedicine addresses both urgent and nonurgent care [17], and that telehealth has become a valuable way to evaluate patients before they enter a medical facility, thereby limiting potential clinician or patient exposure to COVID-19 [18]. Despite the increasing use of telehealth during the COVID-19 pandemic, these studies have not evaluated whether virtual visits actually serve the patient-clinician relationship [16-18]. In addition, studies that have examined patient satisfaction with virtual visits are mostly specialty- or disease-specific [19-21], have small sample sizes or low survey response rates [9,10,21,22], or include only one item to measure patient satisfaction [13].

To gain a more comprehensive understanding of the patient experience during virtual visits, especially patient-clinician engagement in a range of types of virtual visits, we conducted a survey study with patients who received virtual care from a variety of clinicians representing different practices.

## Methods

We conducted a large survey study that included open-ended questions to comprehensively assess patients’ experiences with virtual visits in the Cleveland Clinic Health System. This cross-sectional study was conducted with adults who had a synchronous virtual visit between June 21, 2017, and July 12, 2017. These visits took place on a synchronous video-visit platform developed by the American Well [23]. The study examined scheduled and on-demand virtual visits that used audio and video (telephonic only were not available). With this app, patients can locate and connect with clinicians in an *on-demand* fashion, and clinicians can also schedule visits in advance so that patients can simply click on a link at the designated time to connect directly with their clinician. Visits were termed an *Express Care Online* visit because patients could access a clinician quickly, though still comprehensively receive help. The user interface facilitates virtual visits over smartphones, tablets, and computers [23].

Eligible patients were identified by daily census reports from the previous day’s virtual visits. When patients had more than one virtual visit during study accrual, only the first visit was included. In addition, visits used for clinician training, digital health testing, and those with a duration of 0 minutes were excluded from the study. All 648 eligible patients with visits during that period were sent an email invitation to complete a brief web-based survey regarding their experiences with their recent virtual visit. All nonresponders were sent reminder email invitations and were then called by a member of the research team. Patients were given a US $5 Amazon e-gift card upon completion of the survey. As part of their participation, they were informed about the gift card at the time of recruitment. Given the small amount of compensation, participation bias is likely to be minimal.

Survey items were designed to consider patients as whole persons, evaluate the patient-clinician relationship, and broadly assess patients’ experiences with virtual visits; specifically, we sought to assess patient’s overall satisfaction, overall ease, technical ease, convenience, relationship with the clinician, and perception of clinician empathy. The survey also aimed to evaluate how patients compared virtual visit quality and value with in-person visits. Survey questions were developed after an extensive review of the literature [14,15,24] and several iterations of expert stakeholder input from members of the digital health team, the office of patient experience, and patient volunteers. The survey consisted of 21 items evaluated on a 5-point Likert scale (1=strongly disagree, 2=disagree, 3=neutral, 4=agree, and 5=strongly agree) and text boxes for comments on what patients liked best about their virtual visit and what could be improved. The survey items are presented in Multimedia Appendix 1.

Survey data were collected and managed using the REDCap (Research Electronic Data Capture) electronic data capture system [25]. Survey data were merged with data from the hospital system that included virtual visit characteristics, such as visit type (scheduled or on-demand), device type (computer or mobile device), clinician type (physician or advanced practice provider), clinician specialty, insurance type, and patient demographic information.

First, we computed descriptive statistics. Factor analysis of the 21 survey items revealed three factors: overall satisfaction with the virtual visit (standardized Cronbach α=.93), comfort and ease using virtual visit technology (Cronbach α=.89), and patient-clinician engagement (Cronbach α=.92). Items associated with each factor were summed and dichotomized at the mean for analysis. We used multivariable logistic regression with backward variable selection to assess the associations among patient demographics; virtual visit clinician type and prior patient relationship with their virtual visit clinician; and overall satisfaction with their virtual visit, patient-reported experience using the virtual visit technology, and patient-clinician engagement. Quantitative data were analyzed using SAS 9.4 [26].

For the open-ended questions, a framework analysis of the content was used to identify themes in patients’ narrative comments. Themes were used to create a coding checklist consisting of dichotomous (yes or no) variables. Themes that emerged from comments in response to what patients appreciated about their virtual visit included not having to travel outside home to receive care, enhanced access to care, and convenience. Themes related to recommendations for improvements included enhancements to the user interface and instructions to better prepare for virtual visits. A coding checklist was used to code the text data quantitatively and descriptive statistics were computed.

This study was approved by our medical center’s institutional review board.

## Results

### Overview

Out of the 426 patients who responded to the survey (426/648, 65.7%), nearly two-thirds self-identified as female (273/426, 64.1%). The average age was 46 (range 18-86) years; 70.2% (299/426) of the patients were from Ohio, 6.8% (29/426) were from Florida, 4.2% (18/426) were from Pennsylvania, and 18.7% (80/426) resided in other states. Insurance coverage was undetermined for more than half of the patients (245/426, 57.5%), 23.7% (101/426) had the hospital’s employee health insurance, and 18.7% (80/426) had other private insurance. The demographic characteristics of the respondents were similar to those of the nonrespondents.

A total of 58.4% (249/426) patients had an on-demand virtual visit for an acute concern, and 41.5% (177/426) had a scheduled virtual visit; 28.6% (121/423) of patients knew their virtual visit clinician from a previous in-person appointment with that same clinician, and those patients were older (*P*=.003) and more often male (*P*=.03) compared with patients who did not know their virtual visit clinician. Moreover, most patients who knew their virtual visit clinician had a scheduled rather than on-demand virtual visit (119/121, 98.3%). About 41.8% (178/426) of clinicians were family physicians, whereas the rest represented other physician specialties, and 20.9% (89/426) were advanced practice providers. Most patients used mobile devices such as phones or tablets for their visits (348/424, 82.1%), whereas the rest were connected using a computer. Patient and virtual visit characteristics are presented in Table 1.

**Table 1:** Patient and virtual visit characteristics (N=426).

Characteristics			Value
Age (years), mean (SD)			46 (15.5)
Female, n (%)			273 (64.1)
Ohio resident, n (%)			299 (70.2)
Hospital employee, n (%)			101 (23.7)
**Insurance used, n (%)**			
	Employee Health Plan	101 (23.7)	
	Medical Mutual of Ohio	80 (18.8)	
	Undetermined	245 (57.5)	
Physician virtual clinician, n (%)			336 (78.9)
Prior relationship with clinician (n=423), n (%)			121 (28.6)
**Visit type, n (%)**			
	On-demand	249 (58.4)	

### Overall Satisfaction

The mean overall satisfaction score was 4.4 out of 5 with an SD of 0.78 (see Table 2 for detailed Likert scale survey results). Overall, 81.9% (344/420) of respondents agreed (107/420, 25.5%) or strongly agreed (237/420, 56.4%) that their virtual visit was *as good as* an in-person visit with a clinician. More than half of the respondents agreed (73/417, 17.5%) or strongly agreed (149/417, 35.7%) that their virtual visit was *better than* an in-person visit with a clinician. In multivariable logistic regression, employee patient status (vs nonemployee) was associated with higher odds of overall satisfaction with their virtual visit (odds ratio [OR] 1.9, 95% CI 1.14-3.47). The results from the multivariable logistic regression are presented in Table 3.

**Table 2:** Patient responses to Likert scale survey items (N=426).

Item			Total, n		Strongly disagree, n (%)		Disagree, n (%)		Neutral, n (%)		Agree, n (%)		Strongly agree, n (%)
**Overall satisfaction**													
	My virtual visit made it easy to get the care I needed.	421		6 (1.4)		14 (3.3)		18 (4.3)		94 (22.3)		289 (68.6)	
	For my health concern, my virtual visit was *as good as* an in-person visit with a health care provider.	420		9 (2.1)		27 (6.4)		40 (9.5)		107 (25.5)		237 (56.4)	
	For my health concern, my virtual visit was *better than* an in-person visit with a health care provider.	417		19 (4.6)		49 (11.7)		127 (30.5)		73 (17.5)		149 (35.7)	
	My virtual visit saved me time.	422		9 (2.1)		6 (1.4)		19 (4.5)		62 (14.7)		326 (77.2)	
	My virtual visit was worth the money I spent on the visit.	420		21 (5)		8 (1.9)		46 (10.9)		81 (19.3)		264 (62.9)	
	I would use a virtual visit again.	422		8 (1.9)		7 (1.7)		15 (3.5)		90 (21.3)		302 (71.6)	
	I would recommend a virtual visit to others.	414		9 (2.2)		8 (1.9)		17 (4.1)		82 (19.8)		298 (72)	
**Comfort and ease using virtual visit technology**													
	My virtual visit platform was easy to use.	422		3 (0.7)		13 (3.1)		12 (2.8)		103 (24.4)		291 (68.9)	
	I was comfortable using my virtual visit platform.	423		0 (0)		2 (0.5)		20 (4.7)		105 (25.8)		296 (70)	
	The wait time to see my online health care provider was reasonable.	422		3 (0.7)		4 (0.9)		15 (3.6)		107 (25.4)		293 (69.4)	
	It was easy to see my health care provider during my online visit.	424		7 (1.6)		5 (1.2)		9 (2.1)		91 (21.5)		312 (73.6)	
	It was easy to hear my health care provider during my online visit.	423		9 (2.1)		9 (2.1)		11 (2.6)		92 (21.8)		302 (71.4)	
	It was easy to talk with my health care provider during my online visit.	423		7 (1.6)		6 (1.4)		10 (2.5)		87 (20.6)		313 (74)	
	The technology was easy to use.	423		5 (1.2)		8 (1.9)		18 (4.3)		105 (24.8)		287 (67.8)	
**Patient-clinician engagement**													
	My online health care provider was interested in me as a person.	423		2 (0.5)		2 (0.5)		26 (6.1)		98 (23.2)		295 (69.7)	
	My online health care provider fully understood my health concern.	417		0 (0)		4 (0.9)		14 (3.4)		86 (20.6)		313 (75.1)	
	My online health care provider and I made a plan of action to resolve my health concern.	421		2 (0.5)		6 (1.4)		15 (3.6)		95 (22.6)		303 (72)	
	I believe that the plan of action my health care provider recommended will resolve my health concern.	419		6 (1.4)		8 (1.9)		54 (12.9)		89 (21.2)		262 (62.5)	
	I understand what I need to do next to resolve my health concern.	422		1 (0.2)		3 (0.7)		18 (4.3)		106 (25.1)		294 (69.7)	
	I had enough time with my health care provider during my online visit.	418		1 (0.2)		4 (0.9)		14 (3.3)		102 (24.4)		297 (71)	
	My privacy was respected during my online visit.	415		0 (0)		0 (0)		10 (2.4)		102 (24.6)		303 (73)	

**Table 3:** Predictors of overall satisfaction, patient-clinician engagement, and comfort and ease with virtual technology.

Factor		Odds ratio (95% CI)
**Patient-clinician engagement**		
	Virtual clinician was nurse practitioner or physician assistant (vs family physician)	2.28 (1.25-4.16)
	Employee patient status (vs nonemployee)	1.73 (1.01-2.95)
**Overall satisfaction**		
	Employee patient status (vs nonemployee)	1.9 (1.14-3.17)
	Technical difficulties (vs no technical difficulties)	0.46 (0.28-0.76)
**Comfort and ease using virtual technology**		
	Prior relationship with virtual clinician (vs no prior relationship)	0.58 (0.35-0.98)

Most patients (383/421, 90.9%) reported that their virtual visit made it easy to receive the care they needed. A respondent noted, “It was quick and easy. Instead of finding babysitters for my 4-month-old twins, it was convenient to do right from my home.” Open-ended responses (Table 4) support this finding: patients valued receiving care from their home without traveling to their clinician’s office (145/363, 39.9%) and reported that virtual visits provided convenient access to health care (121/363, 33.3%) and saved them time (98/363, 26.9%). Most patients reported that they would use virtual care again (392/422, 92.9%) and would recommend it to other people (380/414, 91.8%).

**Table 4:** Patient feedback on their virtual visit in their own words.

Domain			Patients, n (%)		Illustrative quotes
**What did you like best about your virtual visit? (n=363)**					
	No travel or stay at home	145 (39.9)		“It was so much better to be able to not have to leave the house when I felt so awful and in so much pain.” [P46]	
	Access or convenience	121 (33.3)		“I used it on a Holiday when my doctor’s office was closed, and it saved me the trip to Urgent Care while in pain.” [P487]	
	Saved time	98 (26.9)		“I saved time, money and my health by being able to have this visit online.” [P109]	
	Easy	94 (25.9)		“Having two kids, it was so easy to face time a provider to get the help I needed.” [P99]	
	Quick	60 (16.5)		“The process was quick.” [P418]	
	Clinician	50 (13.8)		“My online visit was with Dr. [name] and it was an honest pleasure interacting with her. She seemed legitimately interested and concerned regarding my health matter and at no time did I ever feel awkward or rushed.” [P530]	
	Cost	36 (9.9)		“It saved me a lot of time and money. My care and concerns were addressed as if I was visiting the doctor in person. I am very pleased.” [P444]	
	Continuity of care	20 (5.5)		“I loved the fact that I was able to see my provider, and not just anyone. I have seen this doctor for several years, and getting an in-person appointment with her is very hard to do. Therefore, getting an online appointment, I was able to see her faster.” [P82]	
**What can we improve? (n=100)**					
	User interface	30 (30)		“I would say the technology was faulty. I’m not an expert, but I followed the directions and it would not connect us.” [P459]	
	Virtual visit information	14 (14)		“Perhaps a pre-appointment preparation list might help set appropriate expectations for patients. For example, ‘for the following problems, you may need to get additional tests/go to the ER, etc.’ This is because some people may not know these things.” [P96]	
	Insurance coverage	10 (10)		“I would work to partner with more insurance providers to remove those barriers associated with utilizing health care coverage for this service, if I were the management team.” [P307]	
	Access to prescriptions	9 (9)		“I was not able to get my prescription for strep through the express care online.” [P70]	
	Link to the patient portal	5 (5)		“Cross-functionality with MyChart would be great.” [P223]	
	More clinicians	4 (4)		“I want more specialized care in the app. Please have therapists, psychologists and other specialized doctors.” [P51]	

### Comfort and Ease Using Virtual Visit Technology

The mean overall technology experience score was 4.6 out of 5 with an SD of 0.57, and 93.4% (394/422) of patients reported that the virtual visit interface was easy to use. Participants agreed and strongly agreed that they felt comfortable using it (401/423, 94.8%), and most agreed or strongly agreed that the wait times for using it were reasonable (400/422, 94.8%). Respondents could see (403/424, 95.1%), hear (394/423, 93.1%), and talk with (400/423, 94.6%) their clinician easily during their virtual visit. However, 19.9% (84/423) of patients reported that they had technical difficulties during their virtual visit (Table 5), and technical difficulties were associated with lower odds of overall satisfaction among patients (OR 0.46, 95% CI 0.28-0.76) in multivariable logistic regression. In addition, in multivariable logistic regression, having a prior relationship with their virtual visit clinician was associated with less comfort and ease with virtual technology among patients (OR 0.58, 95% CI 0.35-0.98). In open-ended comments, 23.5% (100/426) of patients commented on what could be improved, and 30% (30/100) of these comments recommended improvement of the user interface (see Table 4 for additional patient suggestions for improvement).

**Table 5:** Patient responses to dichotomous survey items (N=426).

Item		Yes; patient, n (%)	No; patient, n (%)
Have you had a previous in-person visit with the health care provider you saw using Express Care Online? (n=423)		121 (28.6)	302 (71.4)
Did you have any technical difficulties during your Express Care Online visit? (n=423)		84 (19.9)	339 (80.1)
**Where would you have gone for medical care if you had not used Express Care Online? (n=426)**			
	Doctor’s Office	229 (53.8)	197 (46.2)
	Urgent Care	112 (26.3)	314 (73.7)
	Emergency Room	21 (4.9)	405 (95.1)
	Retail Clinic	30 (7)	396 (92.9)
	I would not have gone for medical care	50 (11.7)	376 (88.3)

### Patient-Clinician Engagement

Most patients (393/423, 92.9%) agreed that their virtual visit clinician was interested in them as a person. A respondent commented, “The physician was very kind and really listened to my issue and what I had already tried to resolve it” (for additional patient responses, see Table 4). In addition, the patients felt that they were able to work together with their clinicians reciprocally—94.5% (398/421) of respondents reported that together with their virtual visit clinician, they made a plan of action to resolve their health concern. In multivariable logistic regression, a visit with an advanced practice provider (nurse practitioner or physician assistant) was associated with higher odds of patient-clinician engagement compared with visits with a family medicine physician (OR 2.28, 95% CI 1.25-4.16). In addition, employees of our medical center had higher odds of patient-clinician engagement than nonemployees (OR 1.73, 95% CI 1.01-2.95).

The mean patient-clinician engagement score was 4.6 out of 5, with an SD of 0.53. In open-ended comments, patients described the high quality of their virtual visit clinician (50/363, 13.8%), and 95.7% (399/417) of patients agreed that their virtual visit clinician fully understood their health concern. However, some results suggest a need to improve the patient-clinician relationship. Open-ended comments reflected a need for better orientation to virtual visits: 14% (14/100) of respondents recommended that patients be given more information before their virtual visits to know what to expect and how to prepare for their appointment.

## Discussion

### Principal Findings

The large and diverse sample size and high response rate (426/648, 65.7%) suggest robust findings about patient experience of virtual visits as compared with prior studies [27].

This large mixed methods study in a major health system found that patients reported high satisfaction with virtual visits, that the technology was easy to use, and that virtual visits were comparable or better than an in-person visit. However, technical difficulties were associated with lower odds of overall satisfaction among patients (OR 0.46, 95% CI 0.28-0.76) in the multivariable logistic regression. Our findings of high patient satisfaction with virtual visits align with those of other published studies [15,20,28,29]. For example, 95% of patients who participated in a MinuteClinic telehealth visit were very satisfied with the quality of the health care they received and rated telehealth as better than or just as good as a traditional in-person visit [15]. However, our findings are unique and go beyond these other studies because we measured satisfaction in terms of several indicators of patient-clinician engagement. We found that most patients in our study reported high engagement with their virtual visit clinician. There are concerns about the effect of telemedicine on trust-based relationships between patients and clinicians [30] and the ability to express empathy in digital settings [31,32]. Our study suggests that it is possible to measure the patient-clinician engagement and begin to evaluate empathy and collaborative relationships with patients during a virtual visit.

However, both clinician and patient identity influenced the likelihood of strong patient-clinician engagement during virtual visits. Having a pre-existing relationship with the clinician was associated with lower satisfaction. In terms of gender and age, older men who had previous relationships with their virtual visit clinicians were some of the patients that were least satisfied with their experiences. Patients who had a scheduled virtual visit were older than those who had an on-demand visit and were perhaps less comfortable with virtual technology than younger patients. In multivariable logistic regression, having a prior relationship with their virtual visit clinician was associated with less comfort and ease with virtual technology among patients (OR 0.58, 95% CI 0.35-0.98). However, patient age was not a significant predictor of overall satisfaction, patient-clinician engagement, or comfort and ease with virtual technology. Patients who received care from their clinician via a traditional in-person appointment may have experienced discomfort with, or simply did not like, this unfamiliar mode of care with a clinician they know. Gender, age, and the necessity of multiple or repeated virtual visits instead of in-person visits may contribute to less satisfaction and should be explored further in future studies.

In terms of employment and insurance status, employee status was associated with overall satisfaction with virtual visits. In multivariable logistic regression, employee patient status (vs nonemployee) was associated with higher odds of overall satisfaction with their virtual visit (OR 1.90, 95% CI 1.14-3.47). In addition, employees of our medical center had higher odds of patient-clinician engagement than nonemployees (OR 1.73, 95% CI 1.01-2.95). This may be because of benefit coverage for the virtual visit; medical center employees received the care they needed with little to no out-of-pocket costs. Employee patients were not charged for their virtual visit, whereas nonemployee patients were charged up to US $49, depending on insurance coverage. Employee patients may also be more familiar with clinicians, telehealth in general, and virtual visits specifically; thus, their expectations may have been better aligned with the virtual health care they received.

Our study has limitations that need to be considered. Although the sample was diverse in age, gender, and visit and clinician type, a large percentage of patients were from Ohio, insurance was unknown for more than half, and a quarter were employees, all of which may limit the generalizability of our findings. In addition, despite our relatively high response rate, we may have a response bias, but this is unlikely given the similarity of the responders to the nonresponders. Furthermore, we should be cautious not to generalize our findings to all virtual visits, given that this was a nonprobability sample in one health system.

In addition, we do not know the reason for patient visits. We collectively analyzed on-demand and scheduled visits together, although on-demand visits are typically low acuity infections (upper respiratory and urinary tract), whereas scheduled visits are usually more complex conditions where patients have more intense needs [13]. As nearly all patients who had a prior relationship with their virtual visit clinician had a scheduled virtual visit rather than an on-demand one, their expectations for the care they would receive were likely different from those of patients who did not know their clinicians. Furthermore, scheduled and on-demand visits may be fundamentally different. This study suggests that virtual visits may be most satisfactory when used for acute problems or when health care access is otherwise limited rather than nonacute or more complex issues when patients may prefer speaking with their provider in person at a medical facility. Although this study provides an analysis of many kinds of visits, future studies should disaggregate the types of visits and analyze them separately.

Future studies should consider both the quality of these visits and medical outcomes because both are likely to influence patient satisfaction.

In addition, future studies should further explore the range of clinician experience in virtual visits. In multivariable logistic regression, a visit with an advanced practice provider (nurse practitioner or physician assistant) was associated with higher odds of patient-clinician engagement than visits with a family medicine physician (OR 2.28, 95% CI 1.25-4.16). Our finding that patients were more likely to report high engagement with advanced practice providers rather than with family physicians aligns with findings on in-person visits [32] and highlights opportunities to leverage advanced practice providers in telehealth. Future studies should explore the basis of these differences.

Future research and efforts should also focus on the user interface, facilitating patient expectations of the technology, and associations with quality.

### Conclusions

The impact of the virtual interface on patient-clinician relationships is largely unknown, but our findings are encouraging. Our study found that virtual visits facilitate health care access and relationship-building, contributing to satisfying relationship-centered care, a crucial aspect of contemporary patient experiences. Even during a single virtual visit, we found that patients and clinicians could meaningfully engage in relationship-building practices. Strategies to prepare established patients for virtual visits with their clinicians may ease the transition from in-person care to virtual care, resulting in better experiences for both. Patients should be aware of the capabilities and limitations of patient-clinician engagement in virtual visits [33]. Strategies to prepare clinicians for virtual visits would also support a seamless transition to delivering health care virtually (a tip sheet that outlines 10 best practices for communicating effectively with patients during a virtual visit has been provided in Multimedia Appendix 2) [34].

## Appendix

Multimedia Appendix 1 Express Care Online postvisit patient survey.

Multimedia Appendix 2 Top 10 tips for virtual visits: clinician communication.

## Abbreviations

OR: odds ratio
REDCap: Research Electronic Data Capture
