# Supplementary material for: Patient Experience in Virtual Visits Hinges on Technology and the Patient-Clinician Relationship: A Large Survey Study With Open-ended Questions
Source: J Med Internet Res. 2021 Jun 21;23(6):e18488. doi: 10.2196/18488 (PMC8277398; doi:10.2196/18488)
Supplement: Multimedia Appendix 1 [file jmir_v23i6e18488_app1.docx]

**Appendix 1. Express Care Online Post-Visit Patient Survey**

1. How did you connect for your Express Care Online visit? (mobile device, laptop or desktop)
2. Have you had a previous in-person visit with the health care provider you saw using Express Care Online? (yes/no)
3. Where would you have gone for medical care if you had not used Express Care Online? (doctor’s office, emergency room, urgent care, retail clinic, other [specify], I would not have gone for medical care if I had not used Express Care Online)

Please let us know how much you agree with the following statements about your Express Care Online visit by choosing the answer that best reflects your experience (*strongly disagree, disagree, neutral, agree, strongly agree)*.

1. Express Care Online made it easy to get the care I needed.
2. The wait time to see my online health care provider was reasonable.
3. Express Care Online was easy to use.
4. I was comfortable using Express Care Online.
5. It was easy to see my health care provider during my online visit.
6. It was easy to hear my health care provider during my online visit.
7. It was easy to talk with my health care provider during my online visit.
8. The technology was easy to use
9. My online health care provider was interested in me as a person.
10. My online health care provider fully understood my health concern.
11. My online health care provider and I made a plan of action to resolve my health concern.
12. I believe that the plan of action my health care provider recommended will resolve my health concern.
13. I understand what I need to do next to resolve my health concern.
14. I had enough time with my health care provider during my online visit.
15. My privacy was respected during my online visit.
16. For my health concern, Express Care Online was as good as an in-person visit with a health care provider.
17. For my health concern, Express Care Online was better than an in-person visit with a health care provider.
18. Express Care Online saved me time.
19. Express Care Online is worth the money I spent on the visit.
20. I would use Express Care Online again.
21. I would recommend Express Care Online to others.
22. Did you have any technical difficulties during your Express Care Online visit? (yes/no) If yes, please describe (free text).
23. Please let us know what you liked best about your Express Care Online visit (free text).
24. Please let us know what we can improve about Express Care Online (free text).
