# Supplementary material for: Patient Experience in Virtual Visits Hinges on Technology and the Patient-Clinician Relationship: A Large Survey Study With Open-ended Questions
Source: J Med Internet Res. 2021 Jun 21;23(6):e18488. doi: 10.2196/18488 (PMC8277398; doi:10.2196/18488)
Supplement: Multimedia Appendix 2 [file jmir_v23i6e18488_app2.docx]

**Appendix 2. Top 10 Tips for Virtual Visits: Clinician Communication**
